# Implications of regulator of G-protein signaling 5 expression in the pathogenesis of primary and secondary hyperparathyroidism

**DOI:** 10.1186/s12902-022-01066-8

**Published:** 2022-06-09

**Authors:** Xin Li, Yao Lu, Ling Zhang, Aiping Song, Honglei Zhang, Bo Pang, Jun Liu, Xiaoliang Sun, Haoyang Ji, Linping Huang, Meng Yang

**Affiliations:** 1 Institute of Clinical Medicine Research, China-Japan Friendship Hospital, 100029 Beijing, China; 2 Department of General Surgery, China-Japan Friendship Hospital, 100029 Beijing, China; 3 Center of Nephrology, China-Japan Friendship Hospital, 100029 Beijing, China; 4 Department of Pathology, China-Japan Friendship Hospital, 100029 Beijing, China; 5 State Key Laboratory of Infectious Disease Prevention and Control, National Institute for Communicable Disease Control and Prevention, CDC 155, Changbai Road, Beijing 102206 Changping, China

## Abstract

**Objective:**

To study the protein and mRNA expressions of regulator of G-protein signaling 5 (RGS5) in the pathogenesis of hyperparathyroidism.

**Methods:**

The expression of RGS5 protein in 20 primary hyperparathyroidism (PHPT), 31 secondary hyperparathyroidism (SHPT), and 20 control cases were studied by immunohistochemistry (IHC). The expression of RGS5 mRNA in 15 PHPT, 102 SHPT, and 7 normal parathyroid tissue were measured by quantitative real-time PCR (qRT-PCR) method.

**Results:**

The expressions of RGS5 in PHPT tissues were significantly higher than that in SHPT and normal parathyroid tissues (*P* < 0.05). While the differences in RGS5 protein expressions between SHPT and respective control samples were not statistically significant (*P* > 0.05). Likewise, the *RGS5* mRNA expression in PHPT was significantly higher than that in SHPT (*P* < 0.05) and normal parathyroid (*P* < 0.05) samples. In a similar line, the differences in *RGS5* gene expressions between SHPT and control tissues were not statistically significant (*P* > 0.05).

**Conclusions:**

The characteristic RGS5 protein and mRNA levels in hyperparathyroidism might be helpful in discovering the pathomechanism of hyperparathyroidism and novel therapeutic targets as well.

## Introduction

Secondary hyperparathyroidism (SHPT) has been frequently observed in patients with chronic kidney disease, including uremic renal failure. Studies have shown high phosphorus levels, low calcium levels, and vitamin D receptor deficiency are the main etiological factors for developing SHPT [1]. The pathophysiological manifestations of SHPT include parathyroid hyperplasia, elevated parathyroid hormone (PTH)-mediated imbalance in calcium and phosphorus metabolism in the extracellular fluid, osteoporosis, calciphylaxis and adverse cardiovascular events which are associated with the high mortality rate in SHPT [2, 3].

Notably, as for primary hyperparathyroidism (PHPT), parathyroid tumors are the major sites for PTH over-production, and in 80% of the cases, these tumors are sporadic in origin [4]. Pathophysiology of PHPT is reportedly manifested by the increased PTH secretion, high calcium, low phosphorus, decreased calcium sensitivity, osteoporosis, kidney stones, and cardiac diseases [5].

Primary- and secondary HPT have very similar clinical characteristics but follow distinct mechanisms of pathogenesis. Decreased calcium sensitivity plays one of the important roles in the pathogenesis of both PHPT and SHPT; however, their mechanisms of action remain elusive. It has been reported that the regulator of G protein signaling 5 (RGS5) acts as the negative regulator of G protein-coupled receptors and can inhibit calcium-sensing receptor (CaSR) in PHPT [6]. However, there are few studies suggesting the expression of RGS5 in SHPT. In this study, we investigated the difference in the expressions of RGS5 in PHPT and SHPT models to shed light on the pathomechanism of hyperparathyroidism.

## Methods

### Patients

In this study, all tissue specimens were collected from the patients with diagnostically confirmed PHPT or SHPT who underwent parathyroid surgery at the China-Japan Friendship Hospital between 2013 and 2016. Normal parathyroid tissue samples which were accidentally removed during thyroidectomy were collected from control subjects as control specimens. Inclusion criteria for SHPT were emodialysis patients, chronic kidney disease stage 5, entered regular hemodialysis for more than 3 months, 18–75 years old, voluntarily signed the informed consent, elevated levels of intact PTH (iPTH) (> 800 pg/mL), hypercalcemia, hyperphosphatemia, renal osteodystrophy, calciphylaxis, pruritus, and failure to respond to medical therapy. For PHPT, inclusion criteria were primary hyperparathyroidism, higher PTH than normal, hypercalcemia, hypophosphatemia, no secondary factors, 18–75 years old, signed informed consent voluntarily. It`s a retrospective study.

### Immunohistochemistry

The histopathological assessments of nodular hyperplasia glands, parathyroid adenomas, and normal thyroid glands were performed by immunohistochemical (IHC) staining of RGS5 in formalin-fixed paraffin-embedded parathyroid tissue sections of 4-µm thickness from 20 PHPT, 31 SHPT, and 20 control subjects. For IHC, rabbit anti-RGS5 monoclonal antibody (Abcam, ab196799) was used in 1:500 dilution. The tissue sections were stained with the EnVision/HRP method using an automatic immunostainer. Microscopic evaluations of the SHPT and PHPT pathology were carried out under the 20× and 40× objective magnifications. The mean RGS5 expression in each sample was evaluated by measuring the intensity of its expression in roughly 500 cells, including around 100 cells from five different microscopic fields. The intensity of RGS5 expression was scored weak as +, moderate as ++, strong as +++, and very strong as ++++. Observers were kept blind about the sample classification throughout the IHC staining process.

### RNA isolation and quantitative real-time PCR (qRT-PCR)

All the PHPT (15), SHPT (102), and normal (7) parathyroid tissue samples were stored at -80 °C with proper care until the total RNA isolation using TaKaRa MiniBEST Universal RNA Extraction Kit (TaKaRa Biochemicals, Osaka, Japan) following the manufacturer’s instructions. The PirmeScript ™ 1st Strand cDNA Synthesis Kit (TaKaRa Biochemicals) was applied for cDNA synthesis, then qRT-PCR reactions were carried out using SYBR Premix Ex Taq ™ (TaKaRa Biochemicals) and ABI 7500 FAST real-time PCR detection system (ABI, CASRisbad, CA, USA). The RGS5 and internal housekeeping gene’s primer sequences are shown in Table 1. Melting curve analysis was performed to verify the amplicon specificity. The relative mRNA expressions were expressed as 2^-∆Ct^. △ct = RGS5 ct value - β-actin ct value.

### Statistical analysis

The results were statistically analyzed using the SPSS software 17.0. Analysis of variance (ANOVA) and independent sample *t*-test was applied to quantitatively compare the data between multiple groups and between two groups, respectively. Values were expressed as mean ± standard deviation (SD). *P* ≤ 0.05 was considered statistically significant. Normality test and homogeneity of variance test were performed.

## Results

For patients with PHPT, their average age was 53.14 ± 9.45 years. Mean preoperative serum levels were 745 ± 936.7 pg/mL for PTH, 3.09 ± 0.43 mmol/L for Ca^2+^, 0.75 ± 0.18 mmol/L for P, and 183.1 ± 122.41 U/L for ALP. For patients with SHPT, their average age was 49.13 ± 12.03 years. Mean preoperative serum levels were 1802.52 ± 816.4 pg/mL for PTH, 2.65 ± 0.27 mmol/L for Ca2+, 2.27 ± 0.54 mmol/L for P, and 382.75 ± 365.86 U/L for ALP. The pathological diagnosis of SHPT is parathyroid hyperplasia, and the pathological diagnosis of PHPT is parathyroid adenoma.

**Fig. 1:**
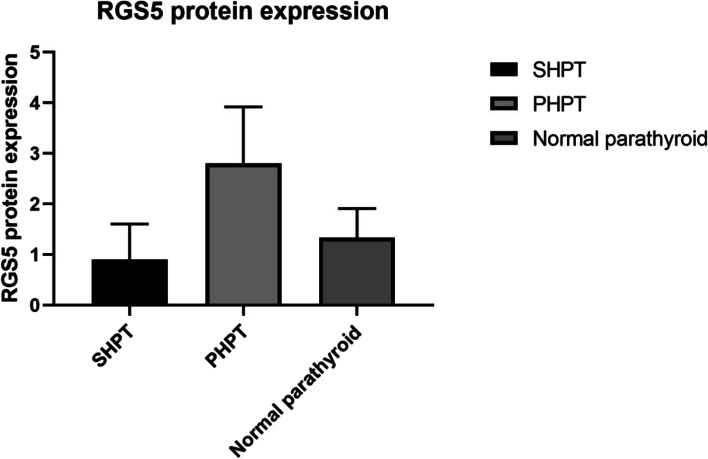
ANOVA test for RGS5 protein expression in different parathyroid tissues, *P < *0.05

**Fig. 2:**
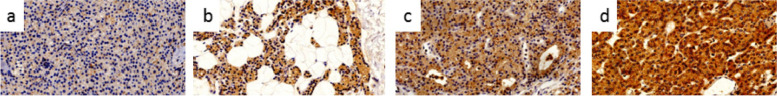
Immunohistochemical staining of RGS5. **a** secondary hyperparathyroidism tissue (0, × 20); **b** normal parathyroid glands (+, × 20); **c** secondary hyperparathyroidism tissue (++, × 20); **d** primary parathyroid glands (+++, × 20)

**Fig. 3:**
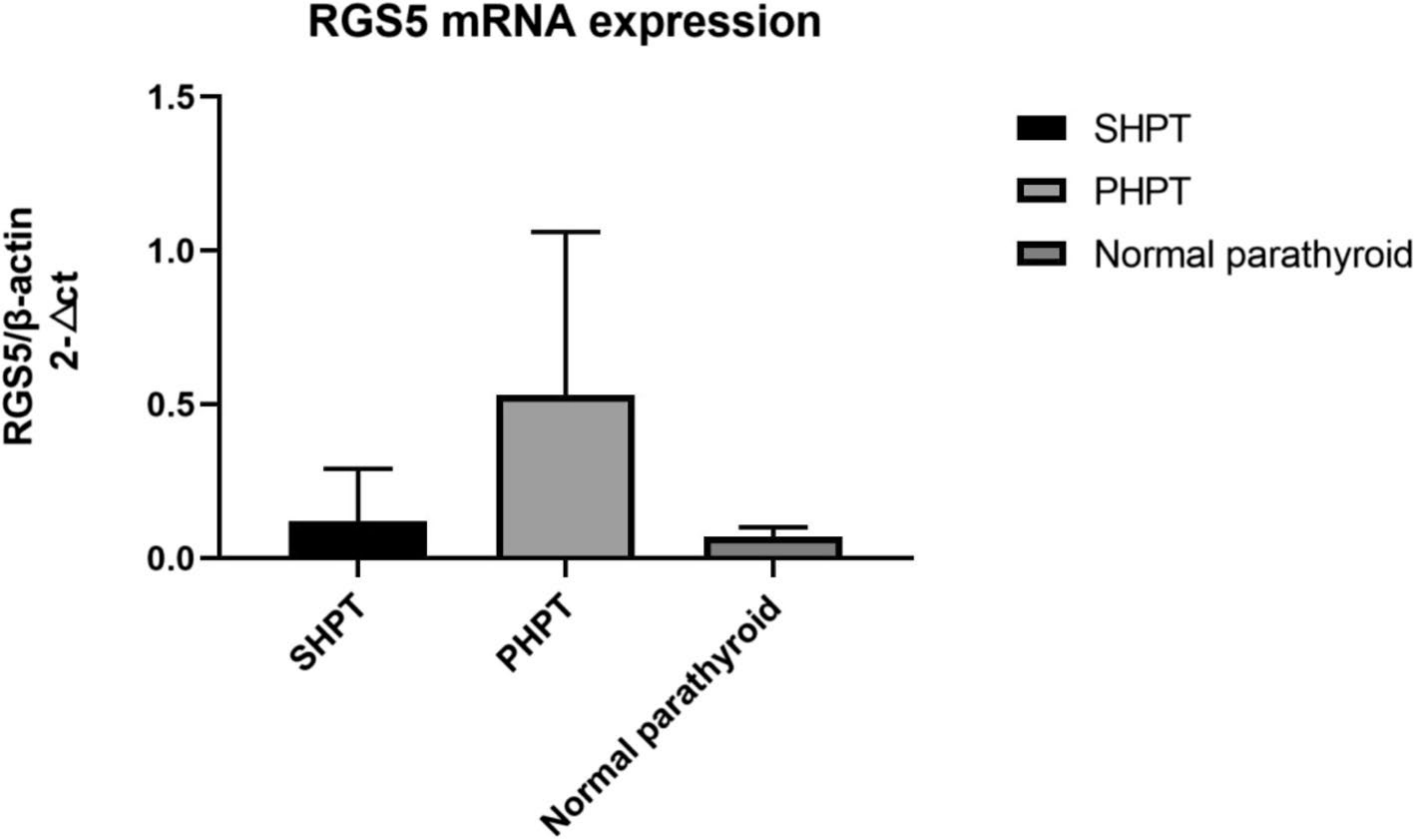
ANOVA test for mRNA expression in different parathyroid tissues (real time PCR 2^−△ct^), *P < *0.05

RGS5 expressions were evaluated in the parathyroid tissue by IHC staining in 51 patients’ samples (20 PHPT and 31 SHPT) and corresponding control samples from 20 subjects. The RGS5 expression in PHPT was significantly higher than that in SHPT (2.8 ± 1.12 vs. 0.9 ± 0.7, *P* = 0.00) and normal parathyroid samples (2.8 ± 1.12 vs. 1.3 ± 0.58, *P* = 0.00). While there was no statistically significant difference in RGS5 expressions between SHPT and control samples (0.9 ± 0.7 vs. 1.3 ± 0.58, *P* > 0.05) (Table 2; Figs. 1 and 2). Furthermore, the RGS5 mRNA expression analysis (117 patients and 7 control subjects) supported its higher protein level in PHPT samples compared to other samples, as there were statistically significant differences in RGS5 mRNA levels between PHPT and SHPT samples (0.53 ± 0.53 vs. 0.12 ± 0.17, *P* = 0.00), and normal parathyroid (0.53 ± 0.53 vs. 0.07 ± 0.03, *P* = 0.00). RGS5 gene expression was highest in cases of PHPT compared to that in other cases. As expected, the difference in RGS5 mRNA expression between SHPT and control samples was not significant (0.12 ± 0.17 vs. 0.07 ± 0.03 *P* > 0.05) (Table 3; Fig. 3).

**Table 1:** Primers used for realtimePCR

Gene	Forward primer	Reverse primer
**RGS5** [6]	TCAGGGCATGGATTCTTTTC	AAGATGGCTGAGAAGGCAAA
**β-actin** [6]	ACTCTTCCAGCCTTCCTTCC	CAGGAGGAGCAATGATCTTG

**Table 2:** ANOVA test for RGS5 protein expression in different parathyroid tissues

	Quantity	Expression value mean ± SD	*P* value
SHPT	31	0.9 ± 0.7	
PHPT	20	2.8 ± 1.12	= 0.00
Normal parathyroid	20	1.33 ± 0.58	

**Table 3:** ANOVA test for CASR mRNA expression(realtimePCR 2^−△ct^)

	Quantity	CASR/β-actin 2^−△ct^ (‾x ± sd)	*P* value
SHPT	102	0.12 ± 0.17	
PHPT	15	0.53 ± 0.53	= 0.00
Normal parathyroid	7	0.07 ± 0.03	

## Discussion

In the present study, we examined the comparative expressions of RGS5 both at protein and mRNA levels between HPT and control samples. Our findings indicated that RGS5 had the highest expression level in the case of PHPT, compared to SHPT and normal parathyroid samples.

RGS family of proteins have been biochemically well-characterized in terms of their inhibitory roles in relaying signals from G-protein-coupled receptor (GPCR) by inducing the dephosphorylation of activated G proteins [7]. RGS5 is an important member of the RGS family, first discovered in 1997, and exhibits constitutive expression in multiple organs, particularly in heart, skeletal muscle, brain, kidney, and liver [8].

It has recently been shown that CaSR expression significantly decreases in the parathyroid glands in the presence of either PHPT or SHPT pathology [9, 10]. CaSR is a 120kD polypeptide having seven membrane-spanning hydrophobic motifs, characteristically resembling the GPCR superfamily proteins. It has been found that CaSR plays a central role in the homeostasis of extracellular calcium concentration through bone turnover, intestinal absorption, and renal reabsorption via the parathyroid gland [11].

In most cases, PHPT pathogenesis is sporadic in nature; however, only 5% of total cases may have a familial origin, such as multiple endocrine neoplasias [12]. Reduced expressions of CaSR and vitamin D (1,25- dihydroxyvitamin D3) receptor (VDR) have been implicated in aberrant calcium signaling in sporadic parathyroid adenomas indicating their mechanistic roles in parathyroid tumorigenesis [13]. Other studies have further shown that CaSR and Myc deregulation may be linked to parathyroid gland tumorigenesis, especially significant downregulation of CaSR in SHPT compared to PHPT adenoma [14].

On the other hand, RGS5 has been found to be overexpressed in some of the parathyroid tumor cases associated with PHPT pathology [6]. In addition, RGS5 can negatively modulate CaSR signaling cascade, thus playing a role in the PHPT pathogenesis [15]. However, the exact function of RGS5 in SHPT pathogenesis has not been explored in detail. We showed that RGS5 was highly expressed in PHPT compared to either SHPT or normal controls. However, between SHPT and normal parathyroid tissues, the difference in the expressions of RGS5 was not statistically significant. Therefore, we speculated that this difference might be related to the differential pathogenesis of PHPT and SHPT. PHPT is associated with an increased set-point for Ca^2 +^ mediated PTH release, which indicates a role for altered CaSR function in the pathogenesis of this disorder. Notably, RGS5 appears to take part in the initial stage of pathogenesis. In the case of SHPT, multiple potent etiological factors like long-term requirement for dialysis, low calcium, and high phosphorus contents, VDR downregulation, fibroblast growth factor 23 (FGF 23) upregulation cumulatively facilitate the disease onset and progression [16]. The altered CaSR function occurs later. During the gradual progression from diffuse hyperplasia to nodular hyperplasia to tertiary hyperparathyroidism stages, the hyperplastic parathyroid glands develop an autonomous secretory system. Perhaps RGS5 is likely to play a role at this stage, which can eventually be a promising research direction in future studies. Our data showed that the *RGS5* gene expression was comparatively higher in SHPT than that in the normal parathyroid tissue, but the difference in expression was not statistically significant. Our results suggest that at the initial stage of transformation into tertiary hyperthyroidism, RGS5 expression follows an increasing trend at the nodular hyperplasia stage. And if this transformation in the hormone secretory system persists, then the difference in RGS5 expression would be significant. Therefore, nodular hyperplasia can be considered as the crucial stage to investigate the mechanism of progression of SHPT to tertiary hyperparathyroidism. Moreover, the RGS5 protein expression in SHPT was found to be lower compared to that in control samples. But the differences in protein levels were not statistically significant. Considering the limitation of the smaller sample size for normal parathyroid samples, the actual difference of protein expression between the normal parathyroid and SHPT has not been shown clearly here.

This study has several limitations. The sample size is not very large, and there is a lack of long-term clinical data follow-up. The analysis of progression from secondary to tertiary hyperparathyroidism is insufficient. The next step is to explore the significance of the difference in RGS5 expression between tertiary hyperparathyroidism and secondary hyperparathyroidism, which is helpful for the study of the pathogenesis of secondary hyperparathyroidism.

## Conclusions

Our research showed that RGS5 might have a significant role in PHPT pathogenesis and progression, possibly via the involvement of CaSR. Hence, downregulation of RGS5 protein expression could be a promising therapeutic strategy for the treatment of PHPT. However, in the case of SHPT, more in-depth research is needed to understand the possible pathogenic effects and therapeutic targets of RGS5.

## Data Availability

The data that support the findings of this study are available on request from the corresponding author. The data are not publicly available due to privacy or ethical restrictions.
